# Community perceptions of mass drug administration for soil-transmitted helminthiasis and schistosomiasis in selected schools in the Philippines

**DOI:** 10.1186/s40249-019-0595-8

**Published:** 2019-10-08

**Authors:** Pauline Joy Lorenzo, Duane Raphael Manzanilla, Dazzle Kane Cortel, Ekaterina Tangog

**Affiliations:** 1 Research Institute for Tropical Medicine, 9002 Research Dr, Alabang Muntinlupa, Manila, 1781 Philippines; 2 University of California - San Francisco, Institute for Global Health Sciences, Mission Hall, Box 1224 550 16th Street, Third Floor, San Francisco, California, 94158 USA

**Keywords:** STH, Schistosomiasis, Deworming, Community perceptions

## Abstract

**Background:**

Soil-transmitted helminthiasis (STH) and schistosomiasis are parasitic infections prevalent in tropical and subtropical countries, such as the Philippines. The prevalence of these infections remain high in certain Philippine provinces, despite established mass drug administration (MDA) programs in endemic communities. This study aimed to understand community knowledge and perceptions of these infections to determine their implications on the current control and elimination strategies, including possible barriers to MDA compliance.

**Methods:**

The study was conducted in Northern Samar and Sorsogon, two provinces with the highest STH and schistosomiasis prevalence in the country. Focus group discussions with separate parent and children groups were utilized to gather knowledge and perceptions on STH and schistosomiasis causes, symptoms, treatment, and prevention; and on the deworming drugs and overall program implementation. Data collection in Northern Samar were done in August 2017, while the sessions in Sorsogon took place in May 2018. A cultural construction of disease framework will show how several factors affect MDA participation.

**Results:**

Results showed that participants held mostly correct biomedical notions of the infections and expressed willingness to participate in MDA program. However, reservations remained due to a reported lack of information dissemination, lack of confidence in the drugs used, and widespread fear of adverse side effects.

**Conclusion:**

Addressing these concerns - improving the conduct of the deworming program, incorporating suggestions from the community, and managing potential adverse events - may help raise MDA participation and encourage better personal preventive practices, reducing STH and schistosomiasis prevalence.

**Trial Registration:**

N/A

## Multilingual abstracts

Please see Additional file 1 for translations of the abstract into the five official working languages of the United Nations.

## Introduction

Soil-transmitted Helminths (STH) are estimated to infect more than 1.5 billion people worldwide, while schistosomiasis places at least 220 million people in need of preventive chemotherapy in 2017 [1–3]. These parasites are prevalent in tropical and subtropical countries, such as the Philippines, with the impoverished identified as the most vulnerable population [4]. Soil-transmitted helminthiasis is caused by different species of intestinal parasites, namely *Ascaris* (*Ascaris lumbricoides*), whipworms (*Trichuris trichiura*), and hookworms (*Ancylostoma duodenale* and *Necator americanus*). *Ascaris* and whipworm are transmitted through ingestion of eggs, while hookworm larvae are hatched in the soil and can cause infection by penetrating unprotected human skin (e.g. those walking barefoot on soil) [1].

Schistosomiasis, on the other hand, is a waterborne infection mainly incurred through contact with parasite larvae in infested bodies of water. The disease is caused by *Schistosoma* parasites (mainly, *Schistosoma haematobium, S. japonicum, and S. mansoni*), whose eggs mature inside certain species of snails [2]. Hence, exposure to fecal-contaminated soil or water due to lack of access to safe water and poor hygiene and sanitation practices promotes the propagation of these intestinal parasites [5].

**Fig. 1:**
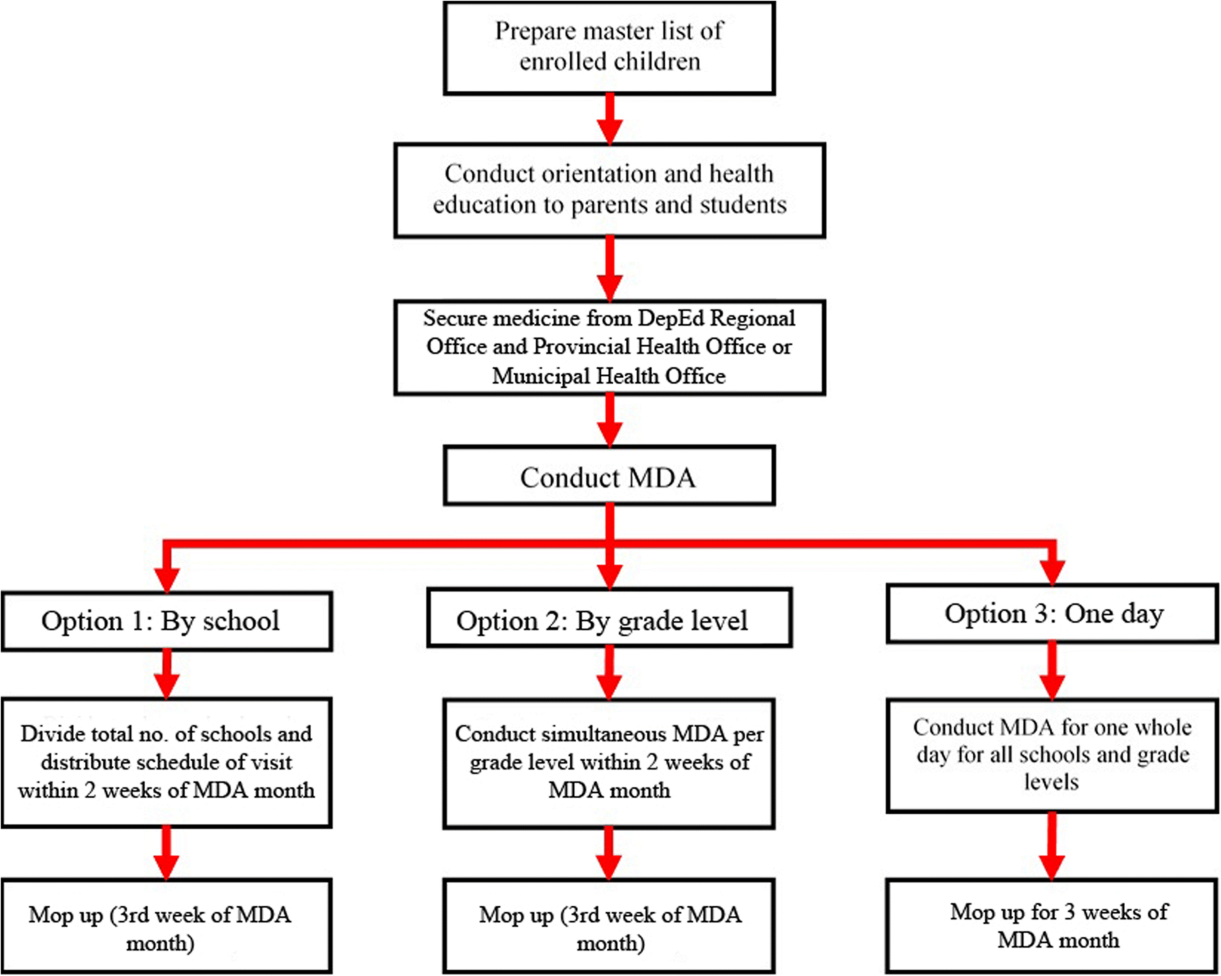
Process of school-based mass drug administration

These parasitic infections are some of the most widespread of the neglected tropical diseases and continue to have significant effects on the physical and cognitive development of school-aged children, pre-school aged children, and women of childbearing age, despite it being treatable [6, 7]. For almost two decades, the Philippines’ Department of Health (DOH) has been employing school-based mass drug administration (MDA) as the general strategy to control STH and schistosomiasis infections in the country. Albendazole (ALB) and mebendazole (MBD) are provided for STH while praziquantel (PZQ) is given for schistosomiasis [8]. MDA activities are conducted bi-annually, usually in public schools, and are led by teachers and principals with the supervision of health workers during the first and third quarter of the school year. The DOH collaborated with the Department of Education (DepEd) to specifically target the most at-risk population: school-aged children [9–11]. Figure 1 illustrates the delivery options for the harmonized MDA for STH and schistosomiasis.

Despite these efforts, the National STH Prevalence Survey conducted by Research Institute for Tropical Medicine (RITM) from 2013-2015 revealed that several provinces in the Philippines still have relatively high prevalence rates of STH infection, and schistosomiasis infections in endemic areas [12]. While the DOH identified poor hygiene practices at home and at school (e.g. open defecation), and lack of access to safe water and sanitation facilities as factors that contribute to the continued transmission of these parasites [13, 14], members of the country’s STH Control Program also cited noncompliance of parents and children in MDA activities as a factor for the consistently high prevalence of infection in some provinces. A study on schistosomiasis control efforts in the Philippines, mentioned the same roadblock, along with lack of attention to non-human reservoirs and insufficient government funding [15]. This suggests that mass drug administration alone cannot eliminate the infection. Most of the published studies conducted in the Philippines about STH and schistosomiasis were prevalence surveys and collections of auxiliary data, such as anthropometric measurements, school performances, dietary intake, and nutritional status in public elementary schools. There is a dearth of information about MDA compliance and factors affecting parent and children participation [5, 7, 16–19].

The main objective of this study was to gain a better understanding of the community perceptions of the STH and schistosomiasis infections, their respective control and prevention practices, and how these perceptions may affect control and elimination programs, particularly in community water, sanitation and hygiene (WASH) practices, and MDA participation. The study also considers that as the primary targets of MDA, children themselves have their own knowledge, attitudes, and practices that must be understood and corrected to help eliminate these infections and thus, must be included in the exploration of community perceptions. A better understanding of these perceptions can provide relevant insights for program managers that will improve current control strategies [20].

## Methods

### Study site

The study was conducted in Northern Samar and Sorsogon, two of the provinces with the highest cumulative STH and schistosomiasis prevalence according to the results of the National STH Prevalence Survey conducted by RITM from 2013 – 2015; with 73.7% and 10%, and 89.5% and 4% STH prevalence and schistosomiasis prevalence, respectively. Study sites were purposively selected based on high prevalence of infection and schistosomiasis endemicity. Only schisto-endemic municipalities were selected as study sites.

In Northern Samar, the municipalities of Catubig, Las Navas, Mondragon, Pambujan, and San Roque were selected as study sites. In Sorsogon, only the municipalities of Irosin and Juban were selected, as they were the only schisto-endemic municipalities in the province.

**Fig. 2:**
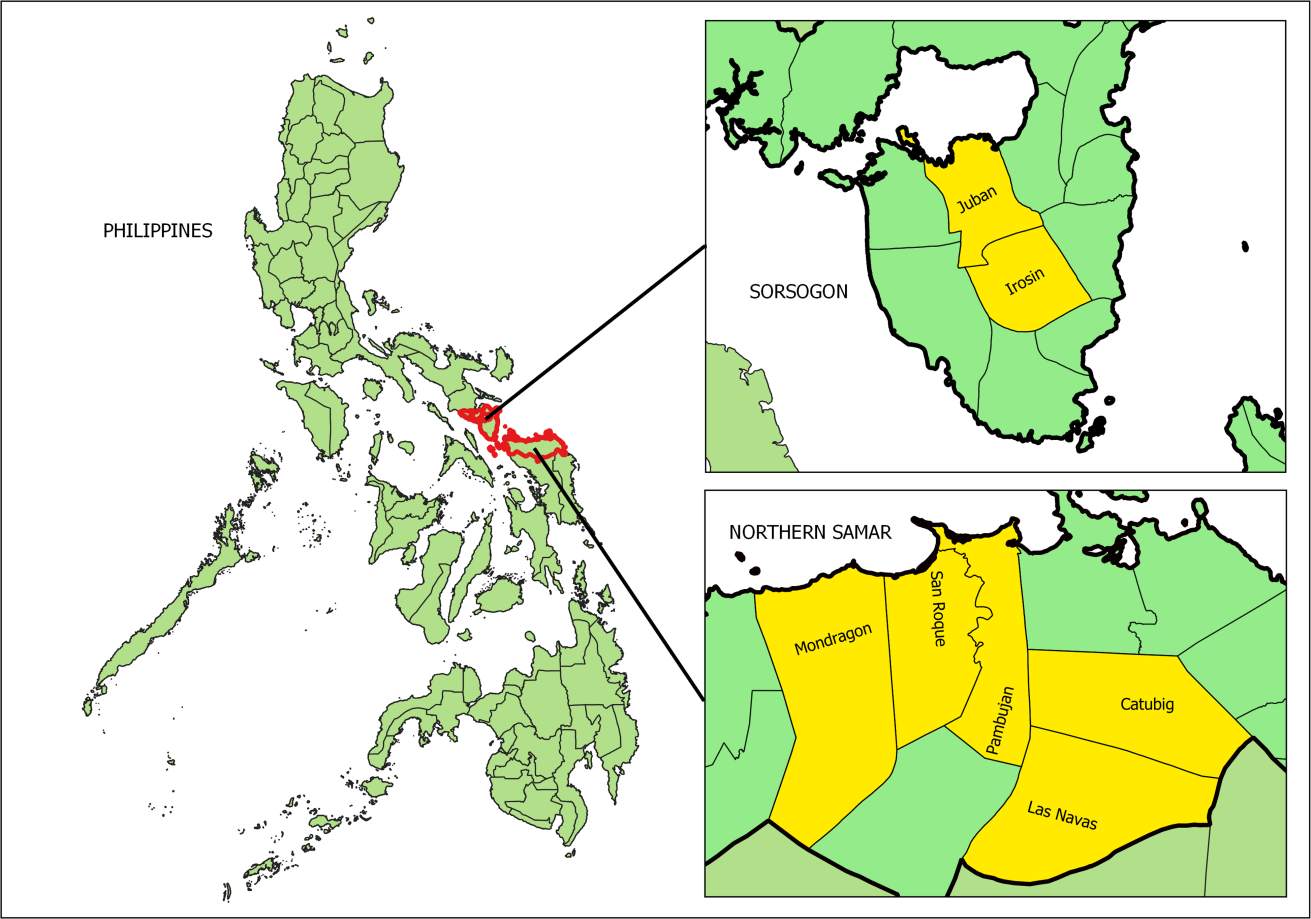
Municipalities selected as study sites

All the selected municipalities were classified as rural communities. Figure 2 shows the location of the selected study sites. Table 1 describes the socio-demographic characteristics of the selected municipalities [21, 22].

**Table 1:** Profile of selected municipalities in Norther Samar and Sorsogon (From PSA Census Data 2010 & 2015)

Municipality	Population (No. of Households)	Primary livelihood	Primary source of water	Primary toilet facility
Mondragon	38 656 (7 593)	Elementary occupation	Piped water	Water-sealed sewer septic tanks
San Roque	30 568 (5 106)	Elementary occupation	Piped water	Water-sealed sewer septic tanks
Pambujan	33 021 (6 115)	Agricultural worker	Shallow well	Water sealed sewer septic tank
Catubig	32 944 (6 514)	Agricultural worker	Shared faucet from community water system	Water-sealed sewer septic tanks
Las Navas	37 896 (7 133)	Agricultural worker	Unprotected spring	Water-sealed sewer septic tank
Irosin	56 615 (11 418)	Elementary occupation	Piped water	Water-sealed sewer septic tanks
Juban	32 175 (6 650)	Elementary occupation	Shared faucet from community water system	Water-sealed sewer septic tanks

### Data collection

Pre-tested semi-structured questionnaires were used to drive Focus group discussions (FGD) with parents/guardians and schoolchildren. Questionnaires were designed to obtain participant knowledge and perceptions on the signs and symptoms, transmission, and treatment of STH and schistosomiasis. Perceptions of the MDA program, specifically, towards the drugs given, perceived benefits and harm, and perceptions on MDA providers were also obtained.

Participants were recruited in partnership with randomly selected public schools in each municipality. A total of ten schools or five per province were invited for this activity. In Northern Samar, one school per municipality was invited for data collection; while in Sorsogon, three schools from the municipality of Irosin and two from Juban were invited. Two sets of FGDs were held per school, one group consisting of parents or guardians and another group consisting of children. Participants were selected using simple random sampling of master lists of students currently enrolled in the Grade VI to Grade VIII levels (10-12 years old). Mothers were invited as the parent participants, because of their usual prominence as primary caretakers in the family, i.e. the assessment of illnesses and subsequent treatment [23, 24].

Coordination with target barangays and schools and pre-testing the data collection tools started in July of 2017. Other pertinent data were gathered during coordination at the Sorsogon site during the data collection activities in May 2018. A total of 20 sessions were conducted. Each activity lasted for 25 to 74 minutes, with an average time of 45 minutes per session. The ten sessions in Northern Samar were conducted in August 2017, facilitated by moderators fluent in the local vernacular (Waray) and a note-taker. The other ten FGD sessions in Sorsogon were conducted from May 21-24 2018. All sessions were held in rooms provided by the principal of each school.

### Data management and analysis

**Fig. 3:**
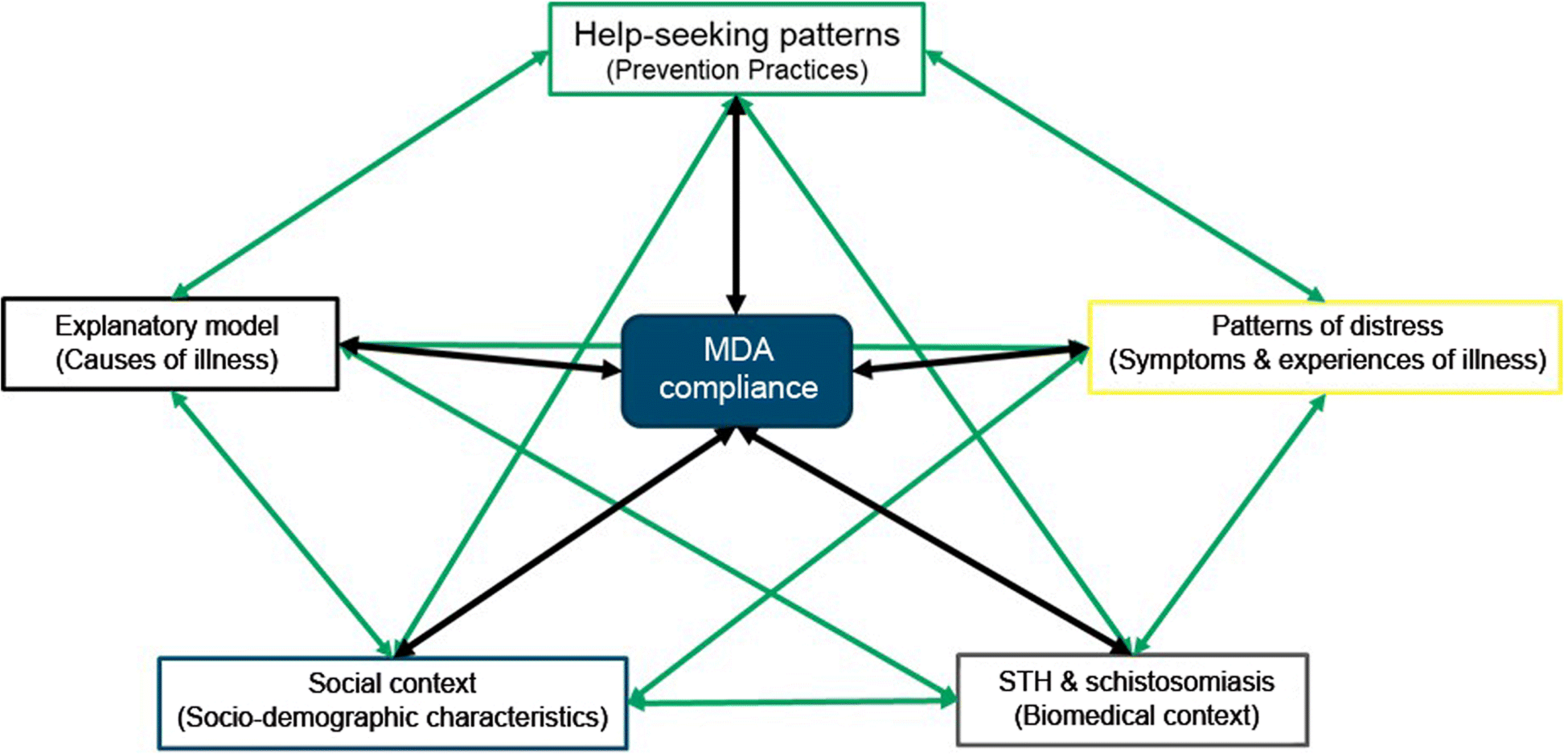
Cultural factors affecting compliance with mass drug administration

All sessions were transcribed verbatim by hired research assistants. Transcripts, field notes, and other recordings were organized and analyzed using the NVivo12^TM^ qualitative data analysis software (QSR International, Melbourne, Australia). For this study, the data were analyzed using thematic framework analysis. Figure 3 shows an adaptation of The Cultural Construction of Diarrheal Illness framework developed by Weiss in 1988 [20] to present how the different set of variables were interrelated in creating a community perception of MDA compliance in selected sites. The framework was used to show how these intestinal parasites, are socially constructed and understood, affecting the MDA participation of parents and children. This provides policy makers and program managers’ opportunities and insights for improving the health education messages delivered and control and elimination strategies implemented in the community.

Following the adapted framework of Weiss [25], there are factors that can affect MDA compliance: patterns of distress, help-seeking behavior, explanatory models, social context, and the STH & schistosomiasis disease. Patterns of distress refer to the symptoms and experiences with the illness. Help-seeking patterns are the practices that people explore to address the distress they endured from the disease. Explanatory models, on the other hand, refer to the meanings and explanations that people put forward to explain their illness. Social context refers to the social, environmental, and even political environment which these people have when they experience the illness. Lastly, the STH and schistosomiasis disease refer to the biomedical explanations, diagnosis, and treatment for the disease. It is important to note that these variables are interconnected, affecting not only MDA compliance, but also the other previously mentioned variables. The framework further allows health practitioners to appreciate the importance of community perceptions of MDA and how it affects their compliance. Moreover, this information is essential in creating innovative ways of improving implementation of MDA by taking advantage of the understanding of how people perceive MDA and how the program works on the ground.

## Results

### WASH practices and conditions

In some FGD sessions, parents claimed that proper handwashing is taught in schools and at home, and that they always remind their children to wash their hands before eating. Nurses and teachers lead handwashing activities in schools, with one participant saying they were taught that handwashing should last for a whole minute. Soap usage is also often cited.

The FGD participants identified artesian wells, rainwater, tap water and filtered water from the river as their sources of water. Faucet water was not potable and was mostly used for washing dishes or bathing. Drinking water was mostly bought from distilling or purifying stations, as some participants pointed out that water from the wells had a reddish tinge, likely due to the rusted pumps.

Most participants declared that they have toilets at home with limited water sources. Some reluctantly admitted to only having modest makeshift latrines using bamboo. Other participants mentioned practicing open defecation. Although the national government provided toilet bowls to the community, some participants were not able to install them, as they still lack the finances to buy other materials for construction. One female participant remarked that *”They (the government) provided the toilet bowl but what about the money for cement? They should provide complete materials, like hollow blocks, and cement”.*. Other participants also recommended that the government provide these materials to allow households to install toilet facilities properly.

### Perceived causes of STH and schistosomiasis

Participants were able to differentiate the causes for each disease. Table 2 summarizes FGD responses to causes of STH and schistosomiasis. Answers regarding sources of STH were more varied, but were still mostly associated with soil and insufficient hygiene practices.

**Table 2:** Focus group discussion responses to perceived causes of STH and schistosomiasis

	STH	Schistosomiasis
Perceived causes	Playing with / in soil	Exposure to dirty or stagnant water
	Walking barefoot	Absence of toilet / Open defecation
	Not washing hands before meals	Exposure to soil
	Dirty nails	Eating fish caught in schisto-infested water
	Consuming junk food	Eating fly-contaminated food
	Eating fly-contaminated food	Eggs
	Eating undercooked food	Snails
	Worms	

For schistosomiasis, exposure to dirty, stagnant water was the most commonly identified source. These refer to still waters found in quarries or rice fields, nonflowing, unlit streams, or any body of water with human or animal stool. Drinking or merely stepping into these waters were said to cause schistosomiasis. Other participants even identified that the water is schisto-infected, if it came from specific areas.

### Perceived symptoms of STH and schistosomiasis

Participants were able to identify similar symptoms and distinguish between other symptoms for both diseases. Table 3 summarizes the perceived symptoms of both STH and schistosomiasis. A malnourished appearance with abdominal distention was recognized as a symptom of both infections, but was the most frequently mentioned sign of STH infection.

**Table 3:** Focus group discussion responses to perceived symptoms of STH and schistosomiasis

	STH	Schistosomiasis
Perceived symptoms	Malnourished appearance	Dizziness
	Enlargement of belly	Fainting
	Stomachache	Foaming at the mouth
	Vomiting	Going crazy/Insanity
	Loss of appetite	Enlargement of belly
	Body aches	Paleness
	Weakness	Blood in stool
	Blurring of eyesight	
	Frequent spitting	
	Anal itching	
	Worms crawling out of body orifices	

Symptoms identified by participants were similar in both study sites. In both provinces, participants seemed to be more aware and fearful of the schistosomal infection of the Central Nervous System. Dizziness, fainting, seizures, foaming at the mouth, and psychosis were frequently cited, due to the parasites purportedly reaching the brain. Although this is more uncommon compared to other types of schistosome infection, this particular type of infection seemed to have made more of an impression in endemic communities, presumably due to the severity and conspicuousness of symptoms. Schistosomiasis was deemed more dangerous than STH infections, because of the possibility of death if left untreated.

### Prevention practices for sTH and schistosomiasis

Table 4 summarizes community perceptions of prevention practices against STH and schistosomiasis. STH prevention mostly included hygiene and sanitation practices such as avoiding soil contact, using toilets for defecation, trimming nails, wearing gloves when handling trash, and avoiding junk food and street food. Taking medicine when needed was also mentioned, and some claimed to self-medicate with herbal medicine or over the counter drugs. Some student participants in Northern Samar also said to avoid salty and sweet food.

**Table 4:** Focus group discussion responses to prevention practices against STH and schistosomiasis

	STH	Schistosomiasis
Prevention practices	Not playing with / in soil	Staying away from rivers and rice fields
	Avoid walking barefoot	Wearing boots
	Improve hygiene and sanitation	Improve hygiene and sanitation
	Take appropriate medicine	Take appropriate medicine
	Avoid eating junk food	Cleaning of surroundings
	Drink only clean water	Avoid consuming dirty, fly-contaminated food

For schistosomiasis, participants also advised overall improvement of hygiene and usage of toilets, with one mentioning that schistosomiasis-infected individuals should have separate toilets from non-infected individuals. Other preventive practices identified were: listening to the community health workers, or barangay health workers (BHWs) as they are locally known, taking appropriate medicine, regular cleaning of surroundings, and not consuming undercooked or dirty food.

### Sources of information

Table 5 summarizes participants’ responses to where they obtained their sources of information for both diseases. The majority of knowledge regarding both diseases were mostly gained from school administrative staff. Seminars and orientations were held to educate the students and the community regarding STH and schistosomiasis.

**Table 5:** Focus group discussion responses to sources of information on STH and schistosomiasis

	STH	Schistosomiasis
Source of information	At school	At school
	Health facility staff	Health facility staff
	Family members	Family members
	Textbooks	

### MDA compliance

**Fig. 4:**
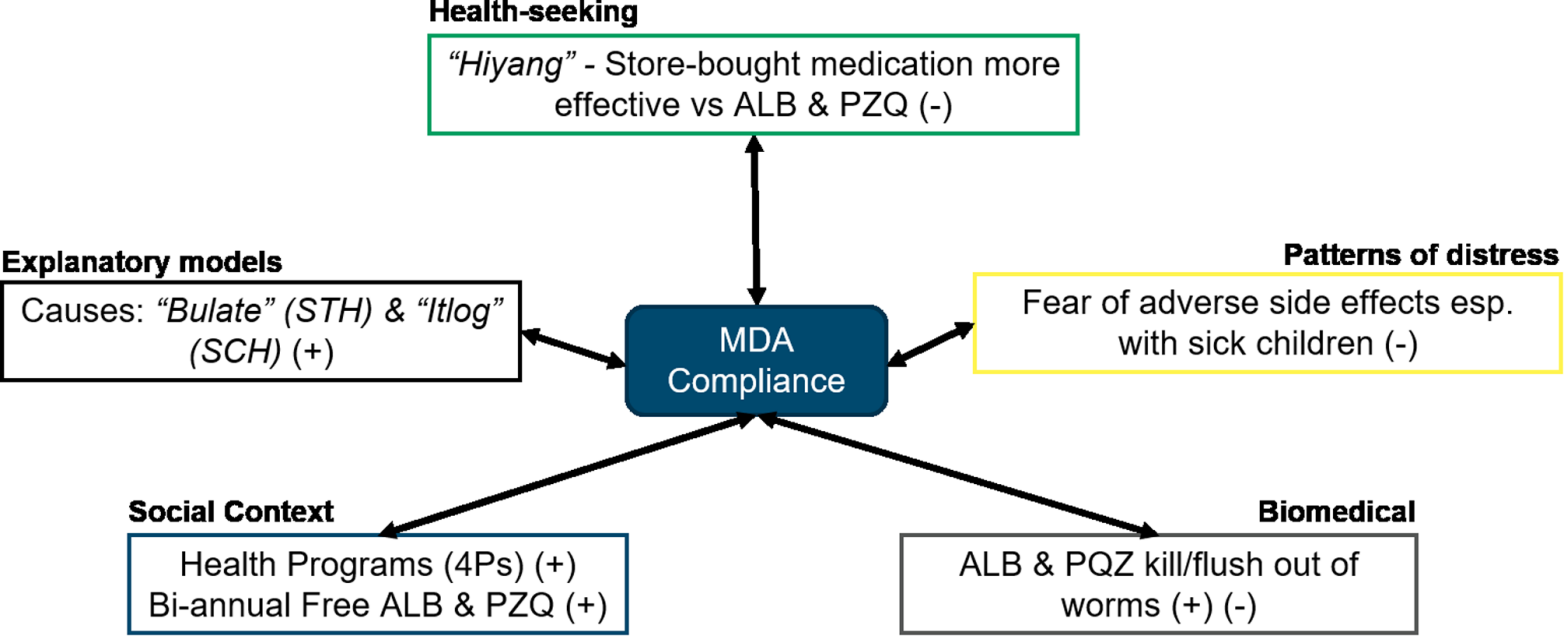
Factors affecting compliance with mass drug administration

Almost all the participants expressed continued willingness to participate in the bi-annual MDA done in schools. Figure 4 showed how several factors contributed to the participation of parents as well as children in the school-based activity. Decrease or elimination of the parasites from their bodies is one of the main reasons for their participation, despite the identified side-effects. Moreover, some parents viewed side effects, such as worms escaping from the body, as a sign that the drugs were effective. A major driver in the partipation rate is compliance with the requirements of the *Pantawid Pamilyang Pilipino Program*, more commonly known as ”4Ps”, a government program that provides conditional cash grants to extremely poor families. One of the conditions for receiving 4Ps grants is the participation of households in MDA activities, ensuring that children receive deworming pills twice a year.

Reasons given for not participating in MDA were mostly medical in nature. Participants, especially the mothers, would refuse signing the consent forms if their children were feverish during the activity. Adverse side effects caused parents and children to hesitate with participation in MDA activities. Side effects identified by children and parents were: dizziness, drowsiness, stomachache, headache, vomiting, increased appetite, fever, loose bowel movement, and rashes. Also prevalent were fears among children that worms would crawl out of their eyes, ears, nose, or mouth. Other participants also expressed doubts over the effectiveness of the pills provided in schools, opting instead to buy purgative medicines from pharmacies or to practice home remedies. Some participants believed that store-bought drugs are more effective than those provided by the national government. Most of the parent participants stated their preference for a certain store-bought drug, due to observations of how worms are flushed from the body after deworming. In one session, some participants shared and agreed that the store-bought drug is more effective, because the worms that came out of their children’s body were all dead, compared to the effects of ALB and MBD, where worms would crawl out alive. One female participant stated *”I feel like the drug (albendazole) does not automatically kill the worms because some would still come out alive. Unlike with Combantrin (the store-bought drug), worms passed with the stool were actually dead.”*

Because of doubts surrounding the effectiveness of deworming drugs and the accompanying side effects, participants wished for better, less troublesome drugs to be administered. In Sorsogon, some participants suggested that the government should provide the store-bought drug brand instead of albendazole and mebendazole due to its perceived efficacy. They also hoped for Praziquantel to be improved, as the drug is widely disliked by children and adults alike due to the side effects, bitter taste, large tablet size, and the need for multiple doses, depending on body weight. Parents also claimed that MDA providers do not check if the drugs are *hiyang* or compatible with their childrens’ constitution. Some parents also suggested that the scope of deworming should be expanded to include whole families, instead of just school children. Current guidelines identify only children up to 18 years of age as the target population for STH deworming [11]. This was suggested as an added measure against the proliferation of parasites.

## Discussion

Overall, people were found compliant towards the school-based deworming activities. There were several factors that affected the participation of people in MDA activities. Responses during the FGD sessions revealed that most of the community perceptions towards the disease adhered to the biomedical explanation of the disease. One of these perceptions is the idea that infections were brought about by causative agents (helminths and their eggs). A quantitative study conducted in Upper Egypt also identified worms as harmful to the children’s health and that there is also a need for treatment [26]. Although rooted in hand to mouth transmission of parasites, persistent erroneous perceptions of disease causation, such as consumption of junk food, sweet food, undercooked, fly-contaminated food need to be corrected. Addressing these will not only change the treatment seeking behavior of the community, but also the preventive measures that they employ within their households. While minimal differences were found between the childrens’ and parents’ knowledge of these infections, childrens’ perceptions need to be consistently taken into account, as they have their own reasons for compliance or noncompliance.

Having a concept of a causative agent that can be treated by ALB and PZQ contributed to the factors that motivate people to participate in MDA activities, despite reported side-effects. However, experiences with these adverse reactions without proper guidance or assistance from MDA providers remained one of the main reasons why they opt to decline in such initiatives at schools, even if they are free of charge. Since this activity is teacher-led, the parents felt that they were not properly trained to handle these adverse reactions, making them hesitant to participate. A study in Guimaras revealed that even the teachers themselves (81% of surveyed teachers) felt ill-equipped to manage possible adverse effects during deworming [27]. However, at the program level, teachers’ assistance is logically and strategically advantageous, as they have the existing infrastructure and administration of the drugs does not required complex skills because of its safety profile [14]. A study conducted in Nigeria expressed the opposite sentiments, where parents preferred school teachers to execute the drug administration, since they were more closer to pupils [28].

Fear of adverse side effects, especially in children, is what mostly dissuades people from participating in deworming. This is consistent with the findings of studies conducted in Tanzania and Uganda [29, 30]. Live worms crawling out from the body was the most dreaded outcome of deworming drugs, adding to the preference for store-bought medication, which were observed to produce less side effects in children. Although they were able to express their doubts and concerns about the effectivity and adverse side-effects of the drugs provided, almost all of participants were still willing to participate in the bi-annual MDA. As stated in the Health Belief Model [31], positive call to action may still take place if the community’s perceived benefits outweigh their perceived barriers towards the activity. In this case, the parents’ knowledge about the ALB and PZQ’s positive effect in their children’s body, and the fact that MDA participation is a requirement for 4Ps outweigh their fears over adverse side-effects. These findings were similar to local studies conducted recently in the provinces of Agusan del sur and Capiz [31, 32].

Conceptions of drug effectiveness may also be important in determining whether people would choose to participate in MDA or self-medicate using over the counter drugs, which were perceived as more effective. FGD sessions revealed that the concept of *hiyang* or ”compatibility” is still widely held, especially by parents, as this plays into the perceived efficacy of medicine and determines whether they will sign the written informed consent forms for the MDA in schools. *Hiyang*, a folk concept wherein persons perceive certain medication as simply compatible or incompatible with their physiology, thereby affecting efficacy [33]. The Tagalog-English dictionaries by Panganiban (as cited in Hardon, 1992)[34], translate the concept as ”being suited, compatible or agreeing usually to medicine, food or company.” A drug that is not *hiyang* for someone is deemed ineffective. Several parents in both study sites speculated that the reason MDA drugs were ineffective for their children was because they were simply not suited to their children’s physiology. Another said that health workers do not assess for this so-called compatibility. Aside from the mere absence of severe adverse reactions from drug intake, children were considered *hiyang* to the drug if the worms turn up dead in children’s stools. Otherwise, parents usually re-evaluated whether they would allow their children to participate in MDA in schools. Nevertheless, ALB and PZQ were still accepted and considered effective by the community. Moreover, being given for free twice a year was appreciated by the community, based on FGD sessions.

The sessions also revealed that other initiatives like the 4Ps incentivize parents to allow their children to take ALB & PZQ. Integrating information about the importance of participating in such activities through other programs might strengthen not only the recall, but the behavior change at the household level. In fact, one of the identified research priorities that has yet to be explored according to the Disease Prevention and Control Bureau or DPCB of the Department of Health is the possible integration of different health service delivery strategies like vaccination and family planning visits [35]. Integration with existing WASH technologies and activities is also a critical factor producing long-term benefits of deterring the continuous transmission of these parasitic infections.

One of the limitations of the study is the absence of quantitative variables that could have determined the level of knowledge, attitude and practice of the community with relation to STH, schistosomiasis and MDA participation. Moreover, conducting similar studies in low prevalence areas will give the researchers a better understanding on the differences of perceptions affecting MDA participation.

## Conclusion

Community knowledge of STH and schistosomiasis transmission were found to be mostly correct. However, there remained misperceptions of disease causation, and an underlying distrust in the drugs used in deworming, particularly in how to handle adverse effects, the capabilities of MDA providers to address them, and perceptions of inferiority to commercially available anthelminthics. Participants recognize this gap in knowledge, leading to vocal requests for better information dissemination and assurances of participants’ safety. These issues could be addressed by strengthening health education messages, and increasing visibility or availability of on-site medical personnel. Understanding how parents and children perceive the MDA activities can help policy makers and program implementors eliminate barriers to MDA compliance, and achieving the target coverage rate for administering chemo-preventive drugs for intestinal parasites.

## Supplementary information

**Additional file 1** Multilingual abstracts in the five official working languages of the United Nations.

## Data Availability

The datasets used and/or analysed during the current study consist mostly of verbatim, untranslated interview and FGD transcripts. Summaries of participants’ answers are available as additional files.

## Abbreviations

ALB: Albendazole
BHW: Barangay health worker
DepEd: Department of Education
DOH: Department of Health
DPCB: Disease Prevention and Control Bureau
FGD: Focus group discussion
IRB: Institutional Review Board
LGU: Local Government Unit
MBD: Mebendazole
MDA: Mass drug administration
NTD: Neglected tropical diseases
PZQ: Praziquantel
RITM: Research Institute for Tropical Medicine
STH: Soil-transmitted helminths
WASH: Water, sanitation and hygiene

## References

[1] WHO. Soil-transmitted helminth infections Fact Sheet. Available from: https://www.who.int/en/news-room/fact-sheets/detail/soil-transmitted-helminth-infections Accessed 26 July 2019.

[2] WHO. Schistosomiasis Fact Sheet. Available from: https://www.who.int/en/news-room/fact-sheets/detail/schistosomiasis Accessed 26 July 2019.

[3] Hotez P, Alvarado M, Basáñez M, Bolliger I, Bourne R, Boussinesq M (2012). The Global Burden of Disease Study 2010: Interpretation and Implications for the Neglected Tropical Diseases. Southeast Asian. J Trop Med Publ Health.

[4] DOH. Guidelines on the Implementation of the National School Deworming Day (DoH AO 2015-0030).

[5] Soares Magalhães R, Salamat M, Leonardo L, Gray D, Carabin H, Halton K (2015). Mapping the Risk of Soil-Transmitted Helminthic Infections in the Philippines. PLOS Negl Trop Diseases.

[6] Brooker S, Mwandawiro C, Halliday K, Njenga S, Mcharo C, Gichuki P (2015). Interrupting transmission of soil-transmitted helminths: A study protocol for cluster randomised trials evaluating alternative treatment strategies and delivery systems in Kenya. BMJ Open.

[7] Ross A, Papier K, Luceres-Catubig R, Chau T, Inobaya M, Ng S (2017). Poverty, dietary intake, intestinal parasites, and nutritional status among school-age children in the rural Philippines. Trop Med Inf Diseases.

[8] DOH. Declaring the Month of July Every Year as the Mass Treatment and Awareness Month for Schistosomiasis in the Established Endemic Areas in the Philippines. 2015. (DoH AO 2009-0013). Manila, Philippines.

[9] DepEd (2007). Implementation of the Mass Deworming Program in All Public Elementary Schools Nationwide (DepEd Memorandum no.28 s.2007).

[10] DOH (2015). Guidelines on the Implementation of the National School Deworming Day (Revised) (DoH AO 2015-0030A).

[11] DOH. Guidelines on the Implementation of the Harmonized Schedule and Combined Mass Drug Administration (HSCMDA) for the Prevention and Control of Lymphatic Filariasis. Schistosomiasis, and Soil-Transmitted Helminths (DOH Memorandum No). 2016;:2016–0212.

[12] RITM-DOH. National Survey on the Prevalence of Soil-Transmitted Helminths (STH), Schistosomiasis and other Intestinal Parasitic Infections among Public School Children (Daycare, Elementary, and High School) in the Philippines: Research Institute for Tropical Medicine; 2016. (Unpublished report).

[13] DOH (2016). Soil-transmitted Helminthiasis Control Program Mass Drug Administration Field Guide.

[14] Belizario V, Tuliao A, Totañes F, Asuncion C (2013). Optimizing school-based intestinal helminth control interventions in the Philippines. Pediatric Infectious Diseases Society of the Philippines Journal (Invited Review).

[15] Inobaya M, Olveda R, Tallo V, McManus D, Williams G, Harn D (2015). Schistosomiasis mass drug administration in the Philippines: Lessons learnt and the global implications. Microbes Inf.

[16] Belizario V, de Leon W, Lumampao Y, Anastacio M, Tai C (2008). Sentinel surveillance of soil-transmitted helminthiasis in selected local government units in the Philippines. Asia-Pacific J Publ Health.

[17] Belizario V, Totañes F, de Leon W, Lumampao Y, Ciro R (2011). Soil-transmitted helminth and other intestinal parasitic infections among school children in indigenous people communities in Davao de Norte, Philippines. Acta Tropica.

[18] Belizario V, Chua P, Liwanag H, Naig J, Erfe J (2014). Soil-transmitted helminthiases in secondary school students in selected sites in two provinces in the Philippines: Policy implications. J Tropical Pedia.

[19] Ng J, Belizario V, Claveria F (2014). Determination of soil-transmitted helminth infection and its association with hemoglobin levels among Aeta schoolchildren of Katutubo Village in Plana, Porac, Pampanga, Philippine. Sci Lett.

[20] Bentley M, Pelto G, Straus W, Schumann D, Adegbola C, de la Pena E (1988). Rapid ethnographic assessment: Applications in a diarrhea management program. Soc Sci Med.

[21] PSA (2010). 2010 Census Housing and Population.

[22] PSA (2015). Census Housing and Population.

[23] Tolhurst R, Amekudzi Y, Nyonator F, Squire S, Theobald S (2008). He will ask why the child gets sick so often: The gendered dynamics of intra-household bargaining over healthcare for children with fever in the Volta Region of Ghana. Soc Sci Med.

[24] Alampay L, Jocson M (2011). Attributions and Attitudes of Mothers and Fathers in the Philippines. Parenting.

[25] Weiss M (1988). Cultural models of diarrheal illness - Conceptual framework and review. Soc Sci Med.

[26] Curtale F, Pezzotti P, Shar Bini A, Al Maadat H, Ingrosso P, Saad Y (1998). Knowledge, perceptions and behaviour of mothers toward intestinal helminths in Upper Egypt: implications for control. Health Policy Plan.

[27] Totanes F, Tuliao A, Ciro R, Macatangay B, Belizario V, Parikh D (2013). Knowledge, attitudes and practices among parents and teachers about soil-transmitted helminthiasis control programs for school children in Guimaras, Philippines. Southeast Asian J Trop Med Publ Health.

[28] Nwaorgu O, Okeibunor J, Madu E, Amazigo U, Onyegegbu N, Evans D (1998). A school-based schistosomiasis and intestinal helminthiasis control programme in Nigeria: acceptability to community members. Trop Med Int Health.

[29] Massa K, Magnussen P, Sheshe A, Ntakamulenga R, Ndawi B, Olsen A (2009). Community perceptions on the community-directed treatment and school-based approaches for the control of schistosomiasis and soil-transmitted helminthiasis among school-age children in Lushoto district, Tanzania. J Biosocial Sci.

[30] Parker M, Allen T, Hastings J (2008). Resisting control of neglected tropical diseases: Dilemmas in the mass treatment of schistosomiasis and soil-transmitted helminths in North-west Uganda. J Biosocial Sci.

[31] Amarillo M, Belizario V, Sadiang-Abay J, Sison S, Dayag A (2008). Factors associated with the acceptance of mass drug administration for the elimination of lymphatic filariasis in Agusan del Sur, Philippines. Parasites Vectors.

[32] Bacon K, Shah M, Taylor L, Macatangay B, Veldkamp P, Belizario V (2012). Assessment of a school-based mass treatment for soil-transmitted helminth infections in Capiz, the Philippines. Southeast Asian J Trop Med Public Health.

[33] Tan M (2008). Revisiting usog, pasma, kulam. n a, editor.

[34] Hardon A (1992). That drug is hiyang for me: Lay perceptions of the efficacy of drugs in Manila, Philippines. Central Issues Anthropol.

[35] Hernandez L. Sustainable Development Goals and DOH Action Plans, National Neglected Tropical Diseases Research Forum. Presentation presented at. Manila Grand Opera Hotel, Manila; 2016.

