# Supplementary material for: Community perceptions of mass drug administration for soil-transmitted helminthiasis and schistosomiasis in selected schools in the Philippines
Source: Infect Dis Poverty. 2019 Oct 8;8:87. doi: 10.1186/s40249-019-0595-8 (PMC6781334; doi:10.1186/s40249-019-0595-8)
Supplement: Supplementary file 1 — Additional file 1 Multilingual abstracts in the five official working languages of the United Nations. [file 40249_2019_595_MOESM1_ESM.pdf]

Translation of the abstract into the five official working languages of the United Nations

تصورات المجتمع حول الإعطاء الجموعي للأدوية الخاصة بالعلاج من داء الديدان الطفيلية وداء البلهارسيا المنتقلتين عن طريق التربة في عدد من المدارس المختارة في الفلبين

بولين جوي لورينزو، دوان رافائيل مانزانيلا، دازل كين كورتيل وإيكاترينا تانوج

ملخص
الخلفية: داء الديدان الطفيلية المنقولة بالتربة (STH) وداء البلهارسيات هما عدوى طفيلية منتشرة في البلدان الاستوائية وشبه الاستوائية، مثل الفلبين. لا يزال معدل انتشار هذه الإصابات مرتفعًا في بعض المقاطعات الفلبينية، على الرغم من برامج الإعطاء الجموعي للأدوية (MDA) في المجتمعات السكنية. وتهدف هذه الدراسة إلى فهم المعارف والتصورات المجتمعية لهذه الإصابات لتحديد آثارها على الاستراتيجيات الحالية لمكافحة العدوى والقضاء عليها، بما في ذلك العقبات المحتملة التي تعترض الامتثال لسياسة الإعطاء الجموعي للأدوية.
المنهج: وقد أجريت هذه الدراسة في شمال سامار وسورسوغون، وهما مقاطعتان تشهدان أعلى معدل انتشار لأدواء الديدان السارية بالتربة وداء البلهارسيا في هذا البلد. تم استخدام مناقشات مجموعة التركيز مع مجموعات منفصلة من الآباء والأمهات والأطفال لجمع المعرفة والتصورات حول الأدواء الديدانية المنقولة بالتربة وأسباب مرض البلهارسيا والأعراض والعلاج والوقاية منه؛ وعلى أدوية التخلص من الديدان والتنفيذ الشامل للبرنامج. تم جمع البيانات في سامار الشمالية في أغسطس 2017، في حين عقدت الجلسات في سورسوغون في مايو 2018. وسوف يبين البناء الثقافي لإطار المرض كيفية تأثير العديد من العوامل على المشاركة في الإعطاء الجموعي للأدوية.
النتيجة: أظهرت النتائج أن المشاركين حملوا في الغالب مفاهيم طبية حيوية حول العدوى وأعربوا عن استعدادهم للمشاركة في برنامج الإعطاء الجموعي للأدوية. ومع ذلك، بقيت التحفظات بسبب نقص نشر المعلومات، وعدم الثقة في العقاقير المستخدمة، والخوف على نطاق واسع من الآثار الجانبية الضارة.
الخاتمة: قد تساعد معالجة هذه المخاوف - في تحسين إدارة برنامج التخلص من الديدان، ودمج اقتراحات من المجتمع، وإدارة الأحداث الضارة المحتملة - في زيادة المشاركة في الإعطاء الجموعي للأدوية والتشجيع على الممارسات الوقائية الشخصية بشكل أفضل، والحد من انتشار أدواء الديدان السارية بالتربة والبلهارسيا.

Translated from English version into Arabic by Bassem Dabas, revised by Arfaoui Firas, through

TRANSLATORS WITHOUT BORDERS

菲律宾部分学校对土源性蠕虫病和血吸虫病大规模群体服药的社区认识

Pauline Joy Lorenzo, Duane Raphael Manzanilla, Dazzle Kane Cortel and Ekaterina Tangog

摘要
引言：土源性蠕虫病(STH)和血吸虫病流行于菲律宾等热带和亚热带国家。尽管在流行社区已经开展了大规模群体服药(MDA)项目，但在菲律宾某些省份，上述疾病的患病率仍很高。本研究旨在了解上述疾病的社区知识和认识，以确定它们对当前控制和消除策略的影响，包括可能影响MDA依从性的障碍。
方法：本研究在菲律宾STH和血吸虫病发病率最高的两个省份——萨马北部和索索贡省进行。通过与不同父母和儿童群体的焦点小组讨论来收集关于STH和血吸虫病的病因、症状、治疗和预防的知识和认识；并就驱虫药物的总体实施方案进行探讨。2017年8月在萨马北部进行数据收集，2018年5月在索索贡举行会议。通过构建疾病框架以了解影响参与MDA的因素。
结果：参与者对上述疾病的生物医学概念基本正确，并表示愿意参与MDA项目。但是，仍然存在保留意见，主要因为缺乏信息沟通和对所使用的药物缺乏信心，且普遍担心药物的不良反应。
结论：解决这些问题——改进驱虫计划的实施方法，采纳社区的建议，管理潜在的药物不良反应事件——可能有

助于提高MDA的参与度，做到更好的个人预防，从而减少STH和血吸虫病的流行。

Translated from English version into Chinese by Xin-Yu Feng, edited by Pin Yang

CHINESE CENTER FOR DISEASE CONTROL AND PREVENTION
NATIONAL INSTITUTE OF PARASITIC DISEASES

Perceptions communautaires de l’administration massive de médicaments contre l’helminthiase transmise par le sol et la schistosomiase dans certaines écoles aux Philippines

Pauline Joy Lorenzo, Duane Raphael Manzanilla, Dazzle Kane Cortel et Ekaterina Tangog

Extrait

Contexte: L’helminthiase transmise par le sol (STH) et la schistosomiase sont des infections parasitaires qui sévissent dans les pays tropicaux et subtropicaux, comme les Philippines. La fréquence de ces infections reste élevée dans certaines provinces des Philipines malgré les programmes d’administration massive de médicaments (AMM) mis en place dans les communautés endémiques. Cette étude a pour but de comprendre la connaissance et les perceptions locales de ces infections afin de déterminer leurs implications sur les stratégies actuelles de contrôle et d’élimination y compris d’éventuels obstacles au respect de l’administration massive de médicaments.

Méthodes: L’étude a été ménée au Samar du Nord et à Sorsogon, deux provinces avec la fréquence la plus élevée de l’helminthiase transmise par le sol et de schistosomiase dans le pays. Les groupes de discussion séparés en groupes de parents et d’enfants ont été organisés à des fins de rassemblement de connaissances et des perceptions sur les causes, les symptômes, le traitement et la prevention de l’helminthiase transmise par le sol et de la schistosomiase ; ainsi que sur les médicaments de vermifugation et la mise en œuvre globale du programme. La collecte des données au Samar du Nord a été effectuée en Octobre 2017, tandis que les séances de Sorsogon ont eu lieu en Mai 2018. Une construction culturelle dans le cadre de la maladie montrera comment plusieurs facteurs affectent la participation à l’administration massive de médicaments.

Résultats: Les résultats ont montré que les participants disposaient pour la plupart des notions biomédicales appropriées, relatives aux infections et exprimaient leur volonté à participer au programme de l’administration massive de médicaments. Cependant, certaines réserves ont été maintenues en raison d’un manque de diffusion de l’information, d’un manque de confiance aux médicaments utilisés et une peur généralisée des effets secondaires indésirables.

Conclusions: Répondre à ces préoccupations – ameliorer le déroulement du programme de vermifugation, prendre en compte les suggestions de la communauté et gérer de potentiels événements indésirables – aidera à augmenter la participation à l’administration massive de médicaments et encouragera l’amélioration des pratiques préventives personnelles, en réduisant ainsi la fréquence de l’helminthiase transmise par le sol et de la schistosomiase.

Translated from English version into French by Loïs Sibefo, revised by Blandine Mathey, through

TRANSLATORS
WITHOUT BORDERS

Представления сообщества о массовом введении лекарств от гельминтоза и шистосомоза, передаваемых через почву, в отдельных школах на Филиппинах

Полин Джой Лоренцо, Дуэйн Рафаэль Мансанилья, Ослепление Кейна Кортеля и Екатерина Тангог

Аннотация

Предпосылки: Гельминтоз, передающийся через почву (СТХ), и шистосомоз - это паразитарные инфекции, распространенные в тропических и субтропических странах, таких как Филиппины. Распространенность этих инфекций остается высокой в некоторых провинциях Филиппин, несмотря на установленные программы массового введения лекарств (МДА) в эндемичных общинах. Это исследование было направлено на то, чтобы понять знания сообщества и восприятие этих инфекций, чтобы определить их влияние на текущие стратегии борьбы и ликвидации, включая возможные барьеры для соблюдения МДА.

Методы: Исследование проводилось в Северном Самаре и Сорсогоне, двух провинциях с самой высокой распространенностью СТХ и шистосомоза в стране. Дискуссии в фокус-группах с отдельными родительскими и детскими группами использовались для сбора знаний и представлений о причинах, симптомах, лечении и профилактике СТХ и шистосомоза; и о противогельминтных препаратах и общей реализации программы. Сбор данных в Северном Самаре был проведен в августе 2017 года, а сессии в Сорсогоне состоялись в мае 2018 года. Культурное построение структуры болезни демонстрирует то, как несколько факторов влияют на участие в МДА.

Результаты: Результаты показали, что участники в основном придерживались правильных биомедицинских представлений об инфекциях и выразили желание участвовать в программе МДА. Тем не менее, оставались неточности из-за сообщения о недостаточном распространении информации, неуверенности в используемых препаратах и широко распространенном страхе побочных эффектов.

Выводы: Решение этих проблем - улучшение поведения программы дегельминтизации, включение предложений от сообщества и управление потенциальными неблагоприятными событиями - может помочь повысить уровень участия в программах по МДА и поощрение улучшенной индивидуальной профилактической практики, снижение распространенности СТХ и шистосомоза.

Translated from English version into Russian by Jurist AKG, revised by Veronika Demeshchyk, through

TRANSLATORS
WITHOUT BORDERS

Percepciones comunitarias de administración masiva de medicamentos, para helmintiasis y esquistosomiasis, las cuales se transmiten por el contacto con suelo, en escuelas seleccionadas de Filipinas

Pauline Joy Lorenzo, Duane Raphael Manzanilla, Dazzle Kane Cortel y Ekaterina Tangog

Resumen

Antecedentes: La helmintiasis transmitida por el suelo (STH, por sus siglas en inglés) y la esquistosomiasis son infecciones parasitarias prevalentes en países tropicales y subtropicales, tales como Filipinas. La prevalencia de estas infecciones sigue siendo alta, en ciertas provincias de Filipinas, a pesar de los programas de administración masiva de drogas que se han establecido (AMD), en comunidades endémicas. Este estudio tuvo como objetivo comprender el conocimiento de la comunidad y las percepciones de estas infecciones, para determinar sus implicaciones, en las estrategias actuales de control y eliminación, incluyendo las posibles barreras para el cumplimiento de MDA.

Métodos: El estudio se realizó en el Norte de Samar y Sorsogon, dos provincias con la mayor prevalencia de STH y

esquistosomiasis, en el país. Se utilizaron de grupos de debate, con grupos separados de padres e hijos para reunir conocimiento y percepciones sobre las causas, síntomas, tratamiento y prevención de STH y esquistosomiasis; y sobre las drogas antiparasitarias y la implementación general del programa. En agosto de 2017 se realizó la recopilación de datos, en el Norte de Samar , mientras que las sesiones, en Sorsogon, tuvieron lugar en mayo de 2018. Una construcción cultural, del marco de la enfermedad, mostrará cómo varios factores afectan la participación de MDA.

Resultados: Los resultados mostraron que los participantes tenían nociones biomédicas, en su mayoría correctas, de las infecciones y expresaron su disposición para participar en el programa AMD. Sin embargo, las reservas se mantuvieron debido a la falta de difusión de información, la falta de confianza en los medicamentos utilizados y el temor generalizado a los efectos secundarios adversos.

Conclusiones: Abordar estas inquietudes (mejorar la conducta del programa de desparasitación, incorporar sugerencias de la comunidad y controlar los posibles sucesos adversos) puede ayudar a aumentar la participación de la MDA y fomentar mejores prácticas preventivas personales, reduciendo la prevalencia de SSTH y esquistosomiasis.

Translated from English version into Spanish by Inmaculada Espárrago, revised by María Luz Puerta, through

TRANSLATORS
WITHOUT BORDERS
